# Genetics of resistance to photobacteriosis in gilthead sea bream (*Sparus aurata*) using 2b-RAD sequencing

**DOI:** 10.1186/s12863-018-0631-x

**Published:** 2018-07-11

**Authors:** Muhammad L Aslam, Roberta Carraro, Anastasia Bestin, Sophie Cariou, Anna K. Sonesson, Jean-Sébastien Bruant, Pierrick Haffray, Luca Bargelloni, Theo H. E. Meuwissen

**Affiliations:** 1 Nofima, P.O. Box 210, N-1431 Ås, Norway; 2 University of Padova, 35020 Legnaro, Italy; 3 SYSAAF, French poultry and aquaculture breeders, 35042 Rennes Cedex, France; 4 FMD, La Brée les Bains, 93100 Montreuil, France; 5 Norwegian University of Life Sciences, 1430 Ås, Norway

**Keywords:** Photobacteriosis, 2b-RAD, Single nucleotide polymorphisms, Linkage mapping, Genome-wide association study, Quantitative trait loci, Genomic selection, Linkage disequilibrium

## Abstract

**Background:**

Photobacteriosis is an infectious disease developed by a Gram-negative bacterium *Photobacterium damselae* subsp*. piscicida* (*Phdp*), which may cause high mortalities (90–100%) in sea bream. Selection and breeding for resistance against infectious diseases is a highly valuable tool to help prevent or diminish disease outbreaks, and currently available advanced selection methods with the application of genomic information could improve the response to selection. An experimental group of sea bream juveniles was derived from a Ferme Marine de Douhet (FMD, Oléron Island, France) selected line using ~ 109 parents (~ 25 females and 84 males). This group of 1187 individuals represented 177 full-sib families with 1–49 sibs per family, which were challenged with virulent *Phdp* for a duration of 18 days, and mortalities were recorded within this duration. Tissue samples were collected from the parents and the recorded offspring for DNA extraction, library preparation using 2b-RAD and genotyping by sequencing. Genotypic data was used to develop a linkage map, genome wide association analysis and for the estimation of breeding values.

**Results:**

The analysis of genetic variation for resistance against *Phdp* revealed moderate genomic heritability with estimates of ~ 0.32. A genome-wide association analysis revealed a quantitative trait locus (QTL) including 11 SNPs at linkage group 17 presenting significant association to the trait with *p*-value crossing genome-wide Bonferroni corrected threshold *P* ≤ 2.22e-06. The proportion total genetic variance explained by the single top most significant SNP was ranging from 13.28–16.14% depending on the method used to compute the variance. The accuracies of predicting breeding values obtained using genomic vs. pedigree information displayed 19–24% increase when using genomic information.

**Conclusion:**

The current study demonstrates that SNPs-based genotyping of a sea bream population with 2b-RAD approach is effective at capturing the genetic variation for resistance against *Phdp*. Prediction accuracies obtained using genomic information were significantly higher than the accuracies obtained using pedigree information which highlights the importance and potential of genomic selection in commercial breeding programs.

**Electronic supplementary material:**

The online version of this article (10.1186/s12863-018-0631-x) contains supplementary material, which is available to authorized users.

## Background

The gilthead sea bream (*Sparus aurata*) is a widely distributed highly important farmed fish species. Global production of gilthead sea bream has risen about 60% during the last decade with an increase of 116,858 to 175,232 metric tons [1]. Mediterranean aquaculture is a major contributor with an annual production of ~ 147,649 metric tonnes [2]. Nonetheless, the industry has challenges ranging from disease outbreaks to consumer acceptance and preferences, as well as fish welfare issues.

Photobacteriosis, which is also known as fish pasteurellosis, is one of the major infectious diseases that causes economic losses [3]. This disease is developed by a Gram-negative bacterium, which may cause high mortalities (90–100%) especially in the larval and juvenile [4, 5] phases. Exploiting the available natural genetic variation and selection for genetic resistance against this infectious agent may prove a highly valuable tool to help prevent or diminish disease outbreaks. Several factors may affect the response to selection, heritability and the index value given to a trait are the more pronounced ones. Rapid genetic progress can be achieved through selective breeding [6] given that the trait is of moderate to high heritability [7]. Recently, it has been reported that resistance to photobacteriosis is moderately heritable with heritability values ranging from 0.18 to 0.45 [8, 9].

RAD-Seq (Restriction-site associated DNA sequencing) is a reduced representation high-throughput sequencing technique for the simultaneous detection of single nucleotide polymorphisms (SNPs) and genotyping of individuals for the detected SNPs [10]. RAD-Seq technique consists of different steps including (a) digestion of genomic DNA with a restriction enzyme, (b) size selection of DNA fragments and library preparation with unique individual specific nucleotide barcodes (c) multiplexing the libraries from different samples, and subsequent high-depth sequencing of the flanking regions. RAD-Seq has already been successfully used in several studies of aquaculture species to generate high-density linkage maps [11–15] and genome wide association studies (GWAS) in a cost-efficient manner [16–18]. 2b-RAD is a flexible and easily-streamlined version of RAD-Seq, which utilizes type IIB restriction enzymes to cleave genomic DNA from both up and downstream of the enzyme recognition site [19]. 2b-RAD is relatively simple and resulting tags are uniform in length, making them ideal for amplification and sequencing on next-generation platforms. It also avoids the sampling error due to its nature to incorporate all the endonuclease recognition sites for sequencing though sampling error may happen in RAD-Seq during size selection step [20]. Several studies have already successfully utilized the 2b-RAD approach for exploring the genetic basis of traits in fish species [9, 21, 22].

The use of recent available technological advancements (i.e. genome-wide sequencing and genotyping technologies) allows to perform high resolution studies to detect molecular markers that are linked to trait(s) of economic importance, which could prove potent tools to overcome challenging genetic correlations. Several studies have indicated that knowledge about genetic markers linked to genes affecting quantitative traits can increase the selection response of animal breeding programs, especially for traits that are difficult to improve by traditional selection [23, 24]. Several studies (genome wide association studies, GWAS) in Atlantic salmon have reported significant association between genetic markers and quantitative traits of economic importance with significant effects of identified loci on trait(s) [25–27].

Moreover, development of genomic resources using described advancements also provides the opportunity to apply advanced selection methods e.g. genomic selection (GS) with state of the art statistical models. The application of GS with genome-wide distributed molecular markers (e.g. SNPs) to breeding is particularly valuable for difficult traits like disease resistance, which is almost impossible to measure on the selection candidates. Breeding values estimated using genomic prediction with genome-wide optimally dense molecular markers can deliver significant improvements in selection accuracy compared to traditional pedigree-based approaches [9, 18, 28, 29].

The current study aimed at identifying genetic basis of resistance to photobacteriosis in the gilthead sea bream by estimating the genetic variation in explaining resistance to this infectious agent, and performing a GWAS to detect quantitative trait loci and estimate accuracy of pedigree vs. genomics based selection.

## Methods

### Population

A population of nearly 1300 gilthead sea bream juveniles originated from the Ferme Marine de Douhet (FMD, Oléron Island, France) breeding nucleus derived from a cross of 109 parents (25 females and 84 males). Parents were crossed by artificial fertilization using full factorial mating design in “blocks” with 8 block in total, and in each block 3–4 females were fertilized by 9–11 males. Finclip tissues were sampled from each parent for later parentage assignment and genomic analysis. All the experimental fish were created the same day and mixed in a single non-sorted batch.

### Challenge test and trait recording

The developed population of nearly 1300 individuals (body weight ~ 3–5 g) at ~ 120 days post hatching was transferred to the experimental aquarium of the Istituto Zooprofilattico Sperimentale delle Venezie (Legnaro, Italy) for the challenge test. The population was kept for 30 days in rectangular fiberglass 4000 L tanks supplied with re-circulating aerated seawater (30 ppt salinity) to get acclimatized and fed with a commercial pellet diet (Neo Supra Al4g, LeGoussant). A total of 1233 fish were challenged with a virulent strain of *Photobacterium damselae subsp. piscicida* (*Phdp,* 249/ITT/99) by intramuscular inoculation of 1000 CFU. The remaining 67 fish represented the biological control group and received intramuscular injection with PBS 0.01 M.

The challenge test lasted for 18 days and mortalities were recorded twice a day within this duration. A random sample of 30 dead fish was analyzed using validated molecular assay detailed by Roberta et al. (2018) [30] to confirm that mortalities were primarily due to the infection. The surviving animals were euthanized with an overdose of MS-222 (100 mg/L) at 18 days post-infection. Two phenotypic traits as a measure of resistance against photobacteriosis were recorded on the tested individuals; dead/survive (*P*_*DS*_) and day to death (*P*_*D*2*D*_) post challenge. Individuals that survived until day 18 were considered to be survivors. Finclip tissue samples were collected from all dead and alive individuals and stored in ethanol 85% at 4 °C.

### DNA extraction, 2b-RAD library preparation and sequencing

Genomic DNA was extracted from ~ 20 mg of collected tissue samples of parents and progeny using a commercial kit (DNA Tissue HTS 96 Kit, Invisorb, Germany), following the manufacturer’s instructions. The resulting DNA quantity was determined by using a Qubit fluorimeter with a dsDNA BR Assay (Invitrogen, California) and DNA quality was checked at 1% agarose gel electrophoresis.

A total of 1342 2b-RAD libraries (109 parents and 1233 juveniles) were prepared following the protocol reported by Wang et al. [19] with minor modifications described as follow. The template DNA for each individual (300 ng) was digested in 6 μl reaction volume using 1.4 U *AlfI* (Thermo Fisher Scientific, USA) at 37° for 1 h, followed by enzyme heat inactivation at 65° for 20 min. The ligation reaction was performed by combining 6 μl of digested DNA with 20 μl of a ligation master mix containing 0.4 μM each of two library-specific adaptors with fully degenerate cohesive ends (5′ -NN- 3′), 10 mM ATP (New England Biolabs, USA), and 1 U T4 DNA ligase (SibEnzyme Ltd., Siberia). Ligation was carried out at 16° for 3 h, with subsequent heat inactivation for 10 min at 65°.

Sample-specific barcodes were designed through a Barcode Generator program (http://comailab.genomecenter.ucdavis.edu/index.php/Barcode_generator). The PCR reaction were prepared in a volume of 50 μl containing mixes of 12 μl of ligated DNA product, 0.5 μM of each primer (P4 and P6-BC, Eurofins Genomics S.r.l, Italy), 0.2 μM each primer (P5 and P7, Eurofins Genomics), 25 mM dNTPs (New England Biolabs, NEB, Ipswich, Massachusetts, USA), 1× Phusion HF buffer, and 1 U TaqPhusion high-fidelity DNA polymerase (New England Biolabs). This PCR reaction of 50 μl was then divided into three independent reactions of 16.6 μl each to reduce the problem of PCR duplicates.

2b-RAD tags were amplified using the following cycling conditions: 98 °C for 4 min, 13 cycles of. 98 °C for 5 s, 60 °C for 20 s, 72 °C for 5 followed by 5 min at 72 °C. Adaptor and primer sequences were those reported in Wang et al. (2012) [19]. The quality of all amplicon libraries was checked at 1.8% agarose gel and then purified using the SPRIselect purification kit (Beckman Coulter, Pasadena, CA). The concentration of the purified libraries was quantified using a Qubit dsDNA BR Assay Kit (Invitrogen, USA) and Mx3000P qPCR Instrument. Additionally, the quality of 10% of randomly selected libraries was also assessed by running them on an Agilent 2100 Bioanalyzer.

Individual libraries were pooled into equimolar amounts by adopting two different multiplexing strategies for parents (64 libraries per pool) and offspring (128 libraries per pool). The quality of each pool was verified on Agilent 2100 Bioanalyzer. Finally, pooled libraries were sequenced on an Illumina NextSeq500 platform (Illumina, San Diego, CA) using 50 base single-end sequencing (v2 chemistry, high output kit - 50 cycles).

### Genotyping RAD alleles

Adapters trimming and filtering based on the quality score of the sequenced reads were performed using custom developed scripts from the 2bRAD pipeline v2.0 [19], with final read length obtained was 36-bp. Reads were filtered out if the average Phred quality score within a sliding window of 4 bp was less than 15. As genomic reference sequence for sea bream was not available, a 2b-RAD tags based reference was developed de novo by clustering the of quality reads of parents. Perl based custom developed scripts in combination with CD-HIT program were used to develop the reference sequence [19, 31] which was further used to call genotypes by aligning individual specific reads to the reference sequence. Alignments of short reads to the reference sequence were performed using bwa samse (V = 0.7.13-r1126) [32]. The mpileup function of SamTools version 1.2 [33] was used to call variants and the call option of bcftools [33] was used to call the genotype at each variant site for each animal.

Genotypes were called for each animal with a minimum genotype quality of 20, a minimum read depth of 5, and a population wise observed minor allele counts for a particular site must be at-least 50. A minimum of 40 individuals in a population needed to have a genotype call that met this criterion at a specific position. A SNP that passed the above mentioned criteria were considered as a putative SNP for further analyses.

### Parentage assignment

The SNP-based genotypic data were available for both parents and offspring, which were further filtered based on polymorphism by applying a criteria of minor allele frequency (MAF ≥ 0.35) and genotyping rate of ≥90%. The remaining selected set of highly informative SNPs (*n* = 750) were then used to construct pedigree applying the likelihood ratio method implemented in CERVUS version 3.0 [34]. Parentage assignments were validated using opposite homozygote count method. [35, 36] with the full set of SNPs.

### Genetic linkage analysis

Quality controls (QC) were performed both at marker and individual level, SNPs were filtered out based on the criteria of locus specific missing rate > 30%, deviation from expected Mendelian segregation (*P* < 0.001), and Hardy-Weinberg equilibrium exact test (*P* < 1.0e^− 7^). Individuals were filtered out based on two criteria; (i) with more than 30% missing rate of genotype calls, and (ii) individuals which had less than 4 full-sibs in a family. The criterion “ii” was only used for the construction of linkage map to include only informative families for analysis and to avoid computational problems in building map. Linkage groups (LGs) were built using a minimum LOD threshold value of 46 in the “SeparateChromosomes” module of Lep-Map v2 [37] by allowing a maximum distance of 20 cM between consecutive SNPs. The “JoinSingles” module of Lep-Map was used to join singular markers to the already defined linkage groups applying LOD score limit of 5 in combination with LOD score difference of 2 between the best LG and the second-best LG of each joined marker. The module “OrderMarkers” was then used to estimate the order and distance between the markers in centiMorgans (cM). “OrderMarkers” implements a hidden Markov model to compute the likelihood of the order of markers [37] . The option “sexAveraged = 1” was applied during execution of “OrderMarkers” when constructing the consensus sex average map. Maps are reported as sex averaged maps unless otherwise indicated.

### Statistical analyses

#### Heritability estimations

A summary of phenotypic data was obtained from a generalized linear model in R. The heritability of *P*_*DS*_ (binary trait dead/survive) and *P*_*D*2*D*_ (day to death) was estimated by ASReml 4.0 [38] using a pedigree (***A***) or genomic (***G***) relationship matrix with the following linear mixed model:

1 $$ \boldsymbol{y}=\boldsymbol{\mu} +\boldsymbol{Zu}+\boldsymbol{e} $$

where ***y*** is a vector of ‘n’ records on *P*_*DS*_ and/or *P*_*D*2*D*_, ***μ*** is an overall mean, ***u*** is a vector of additive genetic effects distributed as $\boldsymbol{u}\sim \boldsymbol{N}\left(\mathbf{0},\boldsymbol{G}{\boldsymbol{\sigma}}_{\boldsymbol{u}}^{\mathbf{2}}\right)$**,** or $\boldsymbol{u}\sim \boldsymbol{N}\left(\mathbf{0},\mathbf{A}{\boldsymbol{\sigma}}_{\boldsymbol{u}}^{\mathbf{2}}\right),$where ${\sigma}_u^2$ is the additive genetic variance, ***G*** and ***A*** are genomic and pedigree relationship matrices, respectively; ***Z*** is the corresponding incidence matrix to additive effects, and ***e*** is the vector of random residual effects with $\boldsymbol{e}\sim \boldsymbol{N}\left(\mathbf{0},\boldsymbol{I}{\boldsymbol{\sigma}}_{\boldsymbol{e}}^{\mathbf{2}}\right)$.

The genomic relationship matrix was constructed using the VanRaden [39] method as $\frac{ZZ^{\prime }}{2\ast {\sum}_{i=1}^{Nsnp}{p}_i\left(1-{p}_i\right)}$; where *p*_*i*_is the allele frequency of second allele and *Nsnp* is the total number of SNP markers, while pedigree relationship matrix was computed using algorithm of Meuwissen and Luo [40]. Heritability (narrow sense) was estimated as the ratio of additive genetic variance to total phenotypic variance by running univariate analyses.

#### Genome wide association analysis

A genome wide association analysis was performed using a linear mixed model equation. The same model as described in (**1**) was used to perform genome wide association analysis, however an additional variable was added to estimate marker effects. The GCTA program [41] with “--mlma-loco” function was used to detect marker ~ trait associations. This approach ensures that the effect of a SNP is estimated by accounting for additive genetic variance captured by all the markers at linkage groups other than the SNP containing linkage group.

SNPs were considered genome-wide significant when they exceed the Bonferroni threshold for multiple testing (alpha = 0.05) of 0.05/*tg*, where *tg* = 22,544 (total number of genome-wide SNPs). The genome-wide significant threshold used in this study was *P* ≤ 2.22 × 10^−06^, which is equivalent to −*log*_10_(*P*) = 5.65.

**Quantile-quantile** plot with distribution of observed vs. expected *p*-values was checked, and the Inflation factor (lambda, λ) was calculated using following equation.

$$ lambda\left(\uplambda \right)=\frac{median\left({\chi}^2\right)}{0.456} $$

#### Estimation of SNP variances

Variances explained by the top significant SNP(s) were estimated using two following approaches (direct and indirect).

1. For the direct approach, variance explained by significant markers was computed as ${Var}_{SNPi}=2{p}_i{q}_i{\alpha}_i^2$ [42]. Therefore, the proportion of the genetic $\left(\%{varG}_{SNP_i}\right)$ or phenotypic ($\%{varP}_{SNP_i}$) variances captured by these markers equals $\frac{{\mathit{\operatorname{var}}}_{SNP_i}}{\sigma_u^2}\times 100$ and $\frac{{\mathit{\operatorname{var}}}_{SNP_i}}{\sigma_p^2}\times 100$, respectively. Where, *p*_*i*_ and *q*_*i*_ are allele frequencies for the major and the minor alleles respectively, *α*_*i*_ is the allele substitution effect, ${\sigma}_u^2$ and ${\sigma}_p^2$ are the genetic and phenotypic variances computed with the above animal model using genomic relationship matrix.
2. For the indirect approach, the proportion of the genetic or phenotypic variance explained by the genome-wide significant (*GWS*) SNP(s) was estimated using the model (**1**) with the addition of a fixed effect of detected *GWS* SNP(s). However, the ***G*** matrix used in this model was constructed with all other SNPs except *GWS* SNP(s). The variance (genetic or phenotypic) explained by the *GWS* SNP(s) was expressed as a reduction in the total genetic or phenotypic variance.

#### Breeding value estimation

Pedigree vs. genomic breeding values for *P*_*DS*_ and *P*_*D*2*D*_ were computed to quantify and compare the accuracy of the breeding values estimated using pedigree or genomic information. The same model (**1**) described under the genome-wide association study was applied and the predictions were performed using PBLUP, GBLUP, BayesB, BayesC [43], and Bayesian Lasso [44] models using the R/BGLR [45] program.

#### Accuracy of prediction

Accuracy of prediction was calculated using cross validation scheme by random masking ~ 20% of the population with 994 training and 248 validation animals.

The mean accuracy of 20 replicates were computed as correlation (*r*_*corr*_) of the estimated breeding value (pedigree/genomic) with the true phenotype, which were scaled by the square root of the heritability as ${r}_{corr}=\frac{\rho \left(G\left[P\right] EBV,y\right)}{\surd {h}^2}$;

where *ρ* = correlation coefficient, G [*P*] *EBV* = represents breeding values estimated using genomic or pedigree information; *y* =observed phenotypes; *h*^2^= genomic or pedigree based heritability estimates.

### Data availability

The information of linkage map and the sequence of tags are available in Additional file 1. The information on potential candidate genes underlying the QTL along with functional description of these candidate genes is available in Additional file 2. The programs used for analyses in this study could either be freely acquired or purchased from relevant developers.

## Results

### Descriptive statistics

**Fig. 1:**
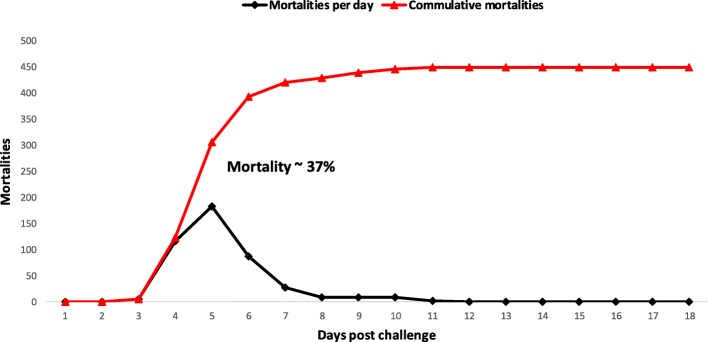
Distribution of mortalities within 18 days of challenge test

Descriptive statistics of the recorded traits (*P*_*DS*_ and *P*_*D*2*D*_) are given in Table 1 with information on recorded traits and available number of observations. Mortality curve and distribution of mortalities along the course of challenge test is presented in Fig. 1. We observed ~ 36.5% of mortality with 449 dead and 784 survivors within 18 days of the challenge test (Fig. 1). The distribution of dead and alive sibs out of total sib count per full-sib family are also plotted which are given in (Additional file 2: Figure S2.1). However, no mortalities were observed in the control group of uninfected fish.

**Table 1:** Descriptive statistics of recorded traits

Traits	N	Missing Values	Mean	Min	Max	SD
*P*_*DS*_	1187	0	0.37	0	1	0.48
*P*_*D*2 *D*_	1187	0	13.28	3	18	6.20

*P*_*DS*_ dead/survive phenotype, binary trait, *P*_*D*2*D*_ day to death phenotype, *N* number of records, *min* minimum values, *max* maximum values, *SD* standard deviation

### Genotyping RAD alleles

Sequencing of 2b-RAD libraries using ~ 14 runs on NextSeq 500 platform yielded a total of 5.21 billion reads, distributed as 0.61 and 4.6 billion across parents and offspring respectively. The mean number of raw reads were 5.59 (±1.55) million per parent and 3.89 (±1.72) million per offspring. The quality filtering of raw reads slightly reduced the number of reads with a 0.50% (26.05 million reads) loss, which resulted in an average number of quality reads of 5.57 million per parent and 3.86 million per offspring.

The catalogue of tags that was built from the quality reads of parents which consisted of 230,500 unique 2b-RAD loci which were used as reference sequence. The SNP calling revealed that 37,247 of the tags had at-least one SNP detected for this population of parents and offspring. However, in order to increase overall informativeness and minimize the amount of missing or erroneous information, SNP data were further filtered using criteria of minor allele frequency ≥ 0.05 per SNP and locus specific genotyping rate of ≥30%, which resulted in 28,330 quality SNPs left for further analyses. Individuals were also removed if genotyping rate of an individual was < 30%, and the level of heterozygosity > 65%. Out of total 1342 individuals, 46 individuals (~ 3%) did not meet this criterion and hence were filtered out. After the above described filtration steps, SNPs based genotyping data consisted of 28,330 loci typed on 1296 individuals (1187 offspring 109 parents).

### Parentage assignment

Pedigree construction using selected highly informative SNP markers produced 177 full-sib families with 1–49 sibs per family. Out of 177 full-sib families, 74 families had a minimum of 5 sibs per family which were used for the construction of linkage map. The comparison of assignment results using likelihood method vs. opposite homozygotes count showed concordance of about 97.79% in assignments.

### Linkage map

The linkage map consisted of 22,544 SNPs, which were grouped into 24 linkage groups (SA01-SA24) with a total sex-average map length of 1970.29 cM (Table 2 and Additional File 1). There were 5786 SNP singleton markers, which did not get assignments to any group. Linkage group SA24 had the lowest while SA02 showed the highest number of SNPs with 758 and 1102 markers respectively. The correlation between number of SNPs and corresponding chromosome map length was 0.549 (*n* = 24 LGs). The female genetic map was 2068.32 cM and the male genetic map was 1727.02 cM.

**Table 2:** Genetic linkage map of gilthead sea bream, *Sparus aurata (SA)*

Linkage Groups	Number of Markers	Male Map Length (cM)	Female Map Length (cM)	Average Length (cM)
SA01	1100	63.09	82.04	79.90
SA02	1102	69.15	89.10	86.68
SA03	997	69.87	85.72	85.98
SA04	1084	89.89	89.82	98.11
SA05	1038	88.11	98.02	103.61
SA06	907	62.22	80.76	78.96
SA07	976	65.65	84.21	81.00
SA08	903	67.01	90.27	84.90
SA09	968	62.48	86.07	81.16
SA10	900	69.77	86.08	77.94
SA11	929	72.00	81.81	77.18
SA12	920	77.39	88.98	83.22
SA13	1003	70.11	87.37	78.22
SA14	904	74.44	90.25	83.77
SA15	912	70.32	91.80	81.30
SA16	876	68.00	81.44	74.57
SA17	880	67.97	80.29	74.75
SA18	912	70.75	83.43	77.96
SA19	941	70.19	82.07	74.84
SA20	893	75.88	89.32	83.08
SA21	918	72.17	99.38	85.01
SA22	868	76.12	87.17	83.42
SA23	855	79.03	76.92	78.83
SA24	758	75.43	76.00	75.88
Total	22,544	1727.02	2068.32	1970.29

The majority of unassigned SNPs showed no-minor homozygote condition, which hints existence of some artifact due to presence of repetitive elements, base call bias etc. In addition, these SNPs were not informative and hence could not be placed in any linkage group. Therefore, all unassigned SNPs were filtered out and the rest of the genetic analyses were performed using 22,544 SNPs.

### Estimates of variance components and GWAS

The estimated variances for *P*_*DS*_ vs. *P*_*D*2*D*_ traits using pedigree and/or genomic information were very similar (Table 3). Both genomic or pedigree based heritability estimates for *P*_*DS*_ and *P*_*D*2*D*_ were ~ 0.32 with genetic correlation of ~ 1.00 indicating that *P*_*DS*_ and *P*_*D*2*D*_ are very similar traits in our dataset possibly due to occurrence of mortalities in a short span (Fig. 1) causing both traits behaving similar.

**Table 3:** Estimates of variance components on dead/survive and days to death phenotype using pedigree vs. genomic information

Components	Pedigree	Genomic
*P*_*DS*_ (${\sigma}_p^2$)	0.239 (0.012)	0.244 (0.012)
*P*_*DS*_ (${\upsigma}_{\mathrm{g}}^2$)	0.0736 (0.020)	0.081 (0.015)
*P*_*DS*_ (h^2^)	0.308 (0.074)	0.332 (0.052)
*P*_*D*2*D*_ (${\sigma}_p^2$)	39.398 (2.003)	39.704 (2.002)
*P*_*D*2*D*_ (${\upsigma}_{\mathrm{g}}^2$)	12.606 (3.426)	12.604 (2.473)
*P*_*D*2*D*_ (h^2^)	0.320 (0.076)	0.317 (0.052))

*P*_*DS*_ dead/survive phenotype, binary trait, *P*_*D*2*D*_ day to death phenotype, ${\sigma}_g^2$ total genetic variance, ${\sigma}_p^2$ total phenotypic variance, *h*^2^ heritability

**Fig. 2:**
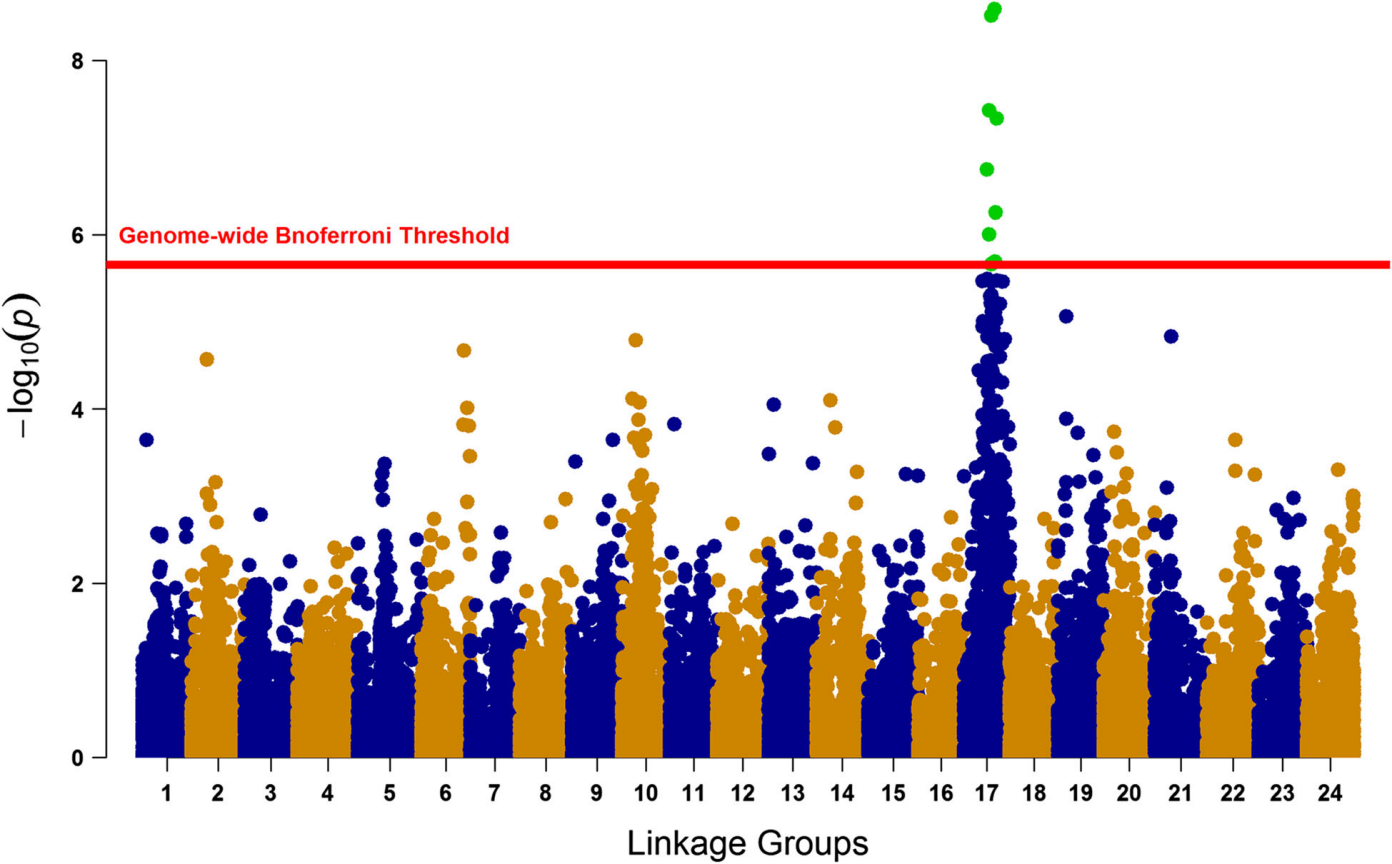
Manhattan plot showing the distribution of –log_10_ *P*-values across linkage groups 1–24

**Fig. 3:**
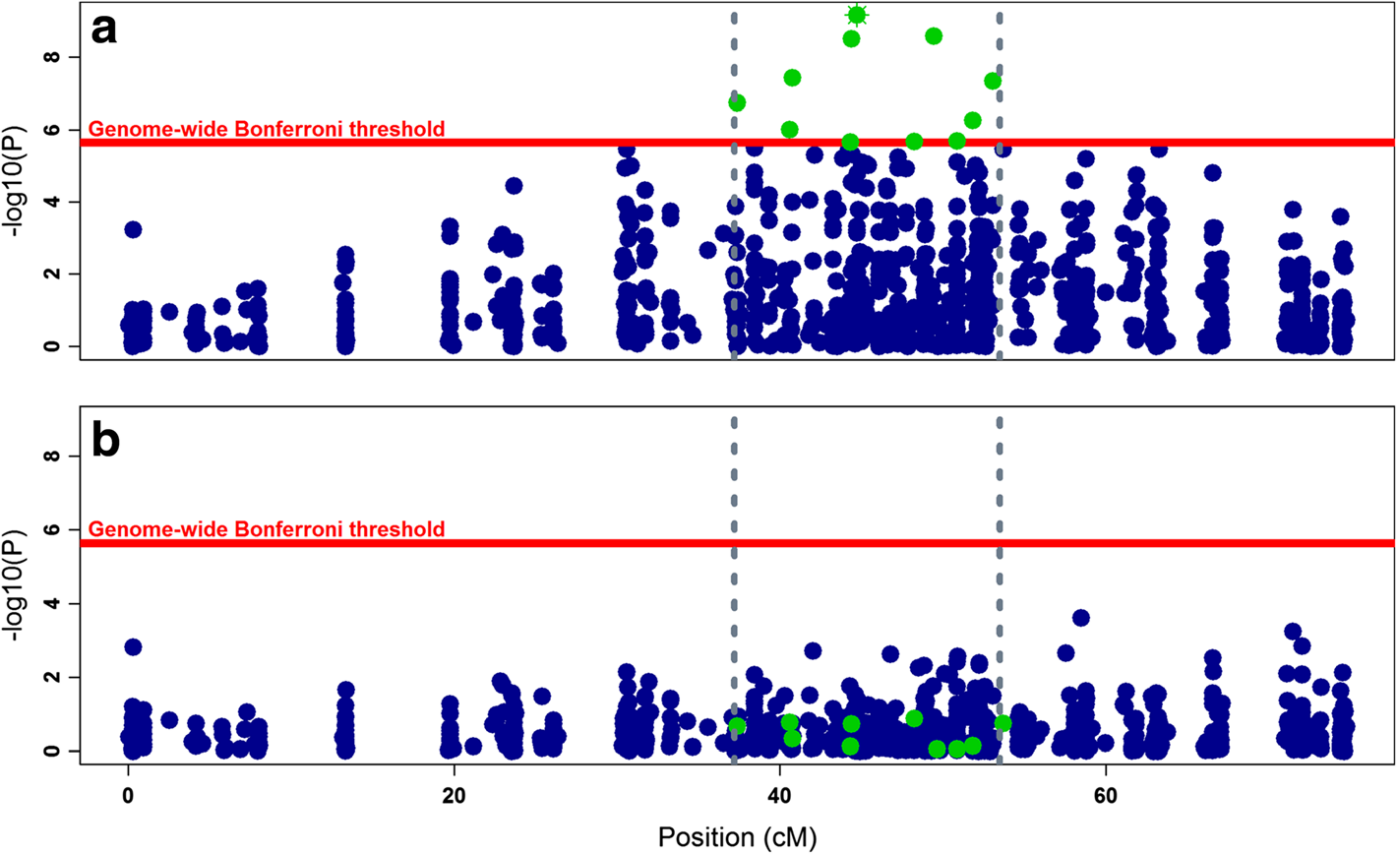
Manhattan plot of –log_10_ *P*-values distributed along the length (cM) of SA17. Highlighted green dots represent genome-wide significant SNPs and horizontal solid line represents the Bonferroni significance threshold (−log_10_ *P*-values = 5.654). **a** Plot with all the markers on SA17 and the top most significant SNP is highlighted green with asterisk (*) symbol; **b** plot after correcting for the top most significant SNP and using it as a fixed effect in the model. Fixing the top most significant SNP in the model caused an overall shrinkage in *P*-values for all the SNPs including previously significant SNPs (highlighted green)

The GWAS analysis for *P*_*DS*_ and *P*_*D*2*D*_ resulted in a clear signal with a strong peak of 11 SNPs at SA17, which surpassed the Bonferroni-corrected genome-wide significance threshold with *p*-value of 2.218e-06 (Fig. 2 and Additional file 2: Figure S2.2). The allele substitution effects, minor allele frequencies and variances explained by these SNPs are presented in table 4. The proportion of total genetic variation captured by the highest significant SNP was 13.28% when computed using the direct method while it was around 16.14% when calculated using indirect method. The detected QTL region (including 11 genome-wide significant SNPs) of SA17 spans from 37.3 to 53.1 cM with peak SNP positioned at 44.7 cM. When the most significant SNP “248936_32” was included as a fixed effect in the GWAS model, none of the 10 surrounding SNPs showed associations with the trait (Fig. 3a-b and Additional file 2: Figure S2.3A-B).

**Table 4:** Variances explained by genome-wide significantly associated SNPs

SA	Locus ID	Pos	Allele1	Allele2	MAF	*α*	SE	*P*-value	%*varG*	%*varP*
17	248936_32	44.729	G	T	0.278	−0.163	0.026	6.69e-10	13.281	4.406
17	24643_19	49.430	A	G	0.467	−0.139	0.023	2.55e-09	11.922	3.955
17	283885_36	44.372	G	C	0.343	−0.140	0.024	3.03e-09	10.929	3.626
17	81173_9	40.766	A	G	0.362	−0.132	0.024	3.74e-08	9.892	3.282
17	211563_28	53.066	A	G	0.353	−0.143	0.026	4.61e-08	11.512	3.819
17	265801_18	37.385	T	C	0.321	−0.132	0.025	1.78e-07	9.457	3.137
17	95842_16	51.835	G	C	0.425	0.116	0.023	5.53e-07	8.113	2.692
17	225775_29	40.582	T	C	0.367	0.122	0.025	9.84e-07	8.570	2.843
17	213907_2	50.873	C	G	0.348	0.114	0.024	2.01e-06	7.278	2.414
17	3001_36	48.242	A	G	0.238	−0.134	0.028	2.09e-06	8.018	2.660
17	53157_6	44.333	T	C	0.179	−0.151	0.032	2.18e-06	8.226	2.729

*SA* linkage group number for gilthead sea bream (*Sparus aurata, SA*), *Pos (cM)* genetic map position of SNP, *A1 & A2* minor & major alleles, respectively, *MAF* minor allele frequency, *α* Allele substitution effect, *SE* standard error, *P* aignificance value, *varG* proportion of genotypic variance explained, *varP* proportion of phenotypic variance explained

Both GWAS and variance components based results for *P*_*DS*_ and *P*_*D*2*D*_ were identical. Hence, GWAS results for only the *P*_*DS*_ are presented and discussed in this main document while GWAS results on *P*_*D*2*D*_ are given in the Additional File 2.

#### Quantile-quantile plot

**Fig. 4:**
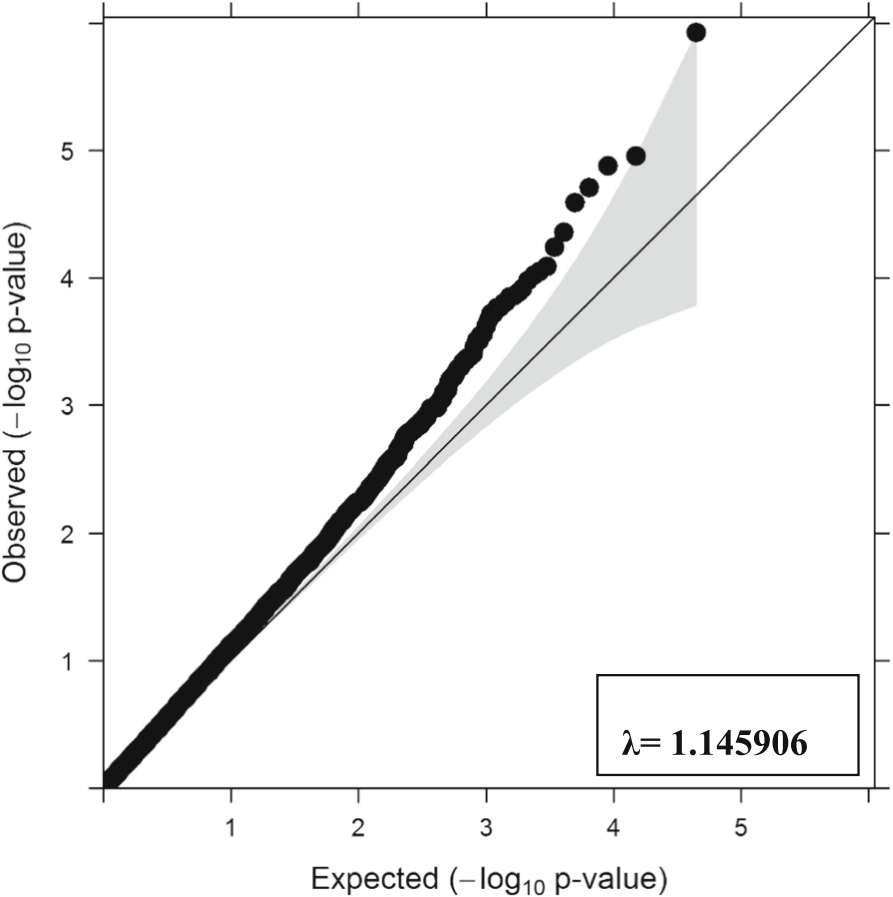
Quantile-quantile plot of –log_10_ *P*-values – dead/survive phenotype (*P*_*DS*_)

A plot presenting distribution of observed vs. expected *p*-values is presented in Fig. 4. The genomic inflation factor (lambda λ) of the fitted GWAS model with all markers was 1.145 and 1.132 (Fig. 4 and Additional file 2: Figure S2.4) for *P*_*DS*_ and *P*_*D*2*D*_ respectively.

### Accuracy of prediction

Prediction accuracies estimated using pedigree and/or genomic information on *P*_*DS*_ and *P*_*D*2*D*_ are given in Table 5. Overall, genomic information based accuracies (GBLUP, BayesB, BayesC, Bayesian Lasso) were significantly higher compared to pedigree information (PBLUP) based accuracy. Average genomic information based accuracies for *P*_*DS*_ and *P*_*D*2*D*_ was 0.569 and 0.545 while pedigree information based accuracy was 0.465 and 0.449 respectively (Table 5).

**Table 5:** Genomic vs. pedigree based prediction accuracies for *P*_*DS*_ and *P*_*D*2*D*_ traits

Model	Accuracy (*P*_*DS*_)	Accuracy (*P*_*D*2*D*_)
PBLUP	0.465 ± 0.098	0.449 ± 0.089
GBLUP	0.559 ± 0.049	0.541 ± 0.043
BayesB	0.577 ± 0.055	0.554 ± 0.043
BayesC	0.567 ± 0.052	0.537 ± 0.035
Bayesian Lasso	0.572 ± 0.048	0.547 ± 0.040

The prediction accuracy for both *P*_*DS*_ and *P*_*D*2*D*_ traits estimated using various genomic information based models showed that the Bayesian models worked better or equally as GBLUP. Accuracy estimates using GBLUP were 0.559 and 0.541 for *P*_*DS*_ and *P*_*D*2*D*_ respectively. In comparison with GBLUP, both BayesB and Bayesian Lasso models yielded marginally improved accuracies for both *P*_*DS*_ and *P*_*D*2*D*_ traits. BayesB model displayed the highest accuracy for both *P*_*DS*_ or *P*_*D*2*D*_ with estimates of 0.577 and 0.554 respectively. For both *P*_*DS*_ and *P*_*D*2*D*_ traits, Bayesian models were ranked (with respect to accuracy) in descending order as BayesB (accuracy = 0.577 and 0.554 respectively), Bayesian Lasso (accuracy = 0.572 and 0.547 respectively) and BayesC (accuracy = 0.567 and 0.537 respectively) with marginal differences in accuracies (Table 5).

## Discussion

The current study aimed at exploring genetic variation contributing resistance against photobacteriosis, detecting QTL(s) for resistance as well as determine the consistency in accuracies for genomic vs. pedigree based prediction methods. Photobacteriosis is one of the major infectious diseases that leads to high economic losses, and it is highly important to find efficient strategies to combat this infectious disease.

The observed mortality of ~ 37% in our challenged test was significantly lower than reported by Antonello et al. [8] and Palaiokostas et al. [9] with more than 95% mortalities. Our experiment used intramuscular inoculation of *Phdp,* which was different compared to the other two studies where fish were infected using bathe method [8, 9]. Observed mortality curve under this model was also discordant from previous reports, which is highly likely due to the method of inoculation and lower dosage concentration (1000 CFU) used in our study. We observed peak mortalities at day 5 post infection, which reached to asymptote around day 8, whereas the studies from Antonello et al. [8] and Palaiokostas et al. [9] showed a much wider distribution of mortalities, and peak mortalities falling between day 7 to 14. The challenge test of our study ultimately produced relatively more variation and an informative mortality curve resulting in a higher proportion of survivors compared to the challenge test of the other two studies where survival was very low.

In the current study, the variances were estimated using two sources of information separately i.e. genomic or pedigree information. Results showed that resistance against *Phdp* (defined as survival after being infected by the pathogen) had moderate heritability with values ranging from 0.308 to 0.332 varying with source of information (pedigree or genomic) and trait (*P*_*DS*_ and/or *P*_*D*2*D*_) used. We did not find any significant difference in heritability estimates of *P*_*DS*_ or *P*_*D*2*D*_, and the genetic correlation between these traits was ~ 1, giving the impression that these are the same trait, possibly because mortalities happened in a very small window of time and hence making both traits behave in the same way. The Moderate heritability illustrates that there is encouraging potential for improving resistance against *Phdp* through selective breeding. The moderate level of heritability estimates (*h*^2^ = 0.31 *to* 0.33) obtained in our study were very similar to those reported by Palaiokostas et al. (2016) [9] (*h*^2^ = 0.22 *and* 0.28), and falling within the range of estimates reported by Antonello et al. (2009) [8] (*h*^2^ = 0.18 *to* 0.45).

In this study, a high-density linkage map was constructed containing 22,544 SNPs distributed over 24 linkage groups, which is consistent with the karyotype of gilthead seabream [46]. The map length obtained in our study was 1970.29 cM, which is slightly longer than what was obtained by Tsigenopoulos et al. (2014) [47], who reported a total map length of 1769.7 cM. The difference in map lengths could be explained by the difference in genome coverage and map resolution. However, the linkage map length obtained by Palaiokostas et al. [9] was twice the map length obtained in this study, which could be due to differences in adopted parameters/methods.

Comparison of sex (male vs. female) specific linkage maps showed an occurrence of sex-biased recombination with total female-specific map was 341.3 cM longer than the male specific map with female-to-male recombination rates of 1.2:1.0 (Table 2). Similar results on sex-specific map lengths were reported by Franch et al. (2006), Tsigenopoulos et al. (2014), and Aslam et al. (2018) [47–49] which was discordant with the results from Palaiokostas et al. [9] who found male specific map (4010 cM) slightly longer (1.05:1.0) than female map (3822 cM). This discordance could be due to differences in populations, and/or different methodological parameters used in both studies. Many fish species including Atlantic salmon, Rainbow trout, Zebrafish etc. have shown significant reduction in recombination rate for the heterogametic sex [50–52]. Similar trend has also been seen in many mammal species e.g. human, dog, pigs etc. [53–55] where heterogametic male expressed lower recombination rate than female. However, sea bream is a protandrous hermaphrodite with ability to produce either kind of gamete (sperm or ova) at different stages of their life, and limited to non is known on sex determination system/loci for this species, and karyotype analysis has not revealed the existence of sex chromosomes [56]. The sex-biased recombination is less likely to reflect occurrence of heterogametic nature in *S. aurata* but possibly progression towards the evolution of sex chromosomes which evolve from autosomes via reduction and/or cessation of recombination, leading to the evolution of heteromorphic sex chromosomes [57].

The genome-wide association analysis yielded a strong peak of 11 SNPs crossing the genome-wide Bonferroni threshold level indicating a QTL at linkage group 17 (Fig. 2 and Additional file 2: Figure S2.2). At-least 8 out of 11 SNPs that crossed genome-wide threshold level in the GWAS analysis showed a favorable effect on the trait with negative *α* values for *P*_*DS*_ and positive *P*_*D*2*D*_, which represent a shift towards zero mortality and increment in surviving days, respectively (Table 4 and Additional file 2: Table S2.5). However, there were 2 to 3 (*P*_*D*2*D*_ *vs*. *P*_*DS*_, respectively) genome-wide significant SNPs that had unfavorable effects on the trait (Table 4 and Additional file 2: Table S2.5) with *α* values in opposite directions which means it would be necessary to find combinations of favorable alleles at those loci to progress the trait in desired direction. The GWAS results from Palaiokostas et al. [9] did not show any genome-wide significant QTL which could be due to differences in genetic background of populations used in both studies and/or the methodological differences of challenge tests used (i.e. route of infection, immersion vs. intramuscular injection, and differences in weight of challenged fish, 0.5-1 g vs. 3–5 g). The distribution of restriction enzyme used for the library preparation becomes much more crucial when populations have different genetic backgrounds as different parts of the genomes might be covered and represented in both studies.

The obtained λ values of GWAS for *P*_*DS*_ and *P*_*D*2*D*_ were 1.145 and 1.132 respectively (Fig. 4 and Additional file 2: Figure S2.4), which was slightly inflated from the acceptable limit of 1.1 [58] indicating small inflation in *P*-values of GWAS. We tested 5–10 principal components for the population structure/stratification but did not find any significant effect on λ coefficient. This slight inflation could be due to an initial variation of body weight (~ 3–5 g) in the challenged population which could not be adjusted due to lack of individual specific records on body weight.

**Fig. 5:**
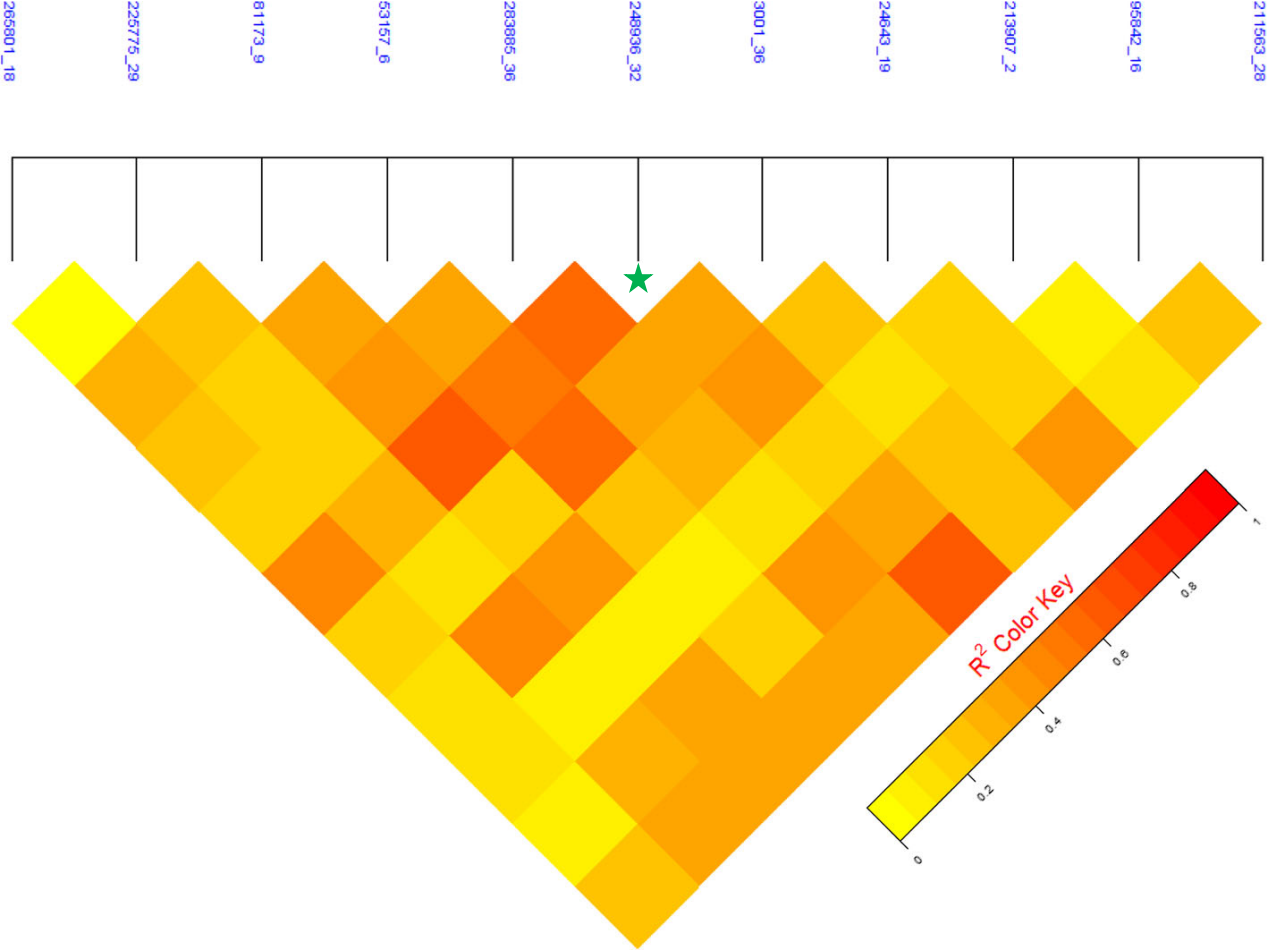
Heatmap of LD structure among genome-wide significant SNPs of GWAS analysis. Locus ID with green asterisk sign represents the top most significant SNP

Variances explained by the top significant SNPs of GWAS were estimated using two methods, the direct method estimated SNP specific variances from the *α* and allele frequencies [59], and the indirect method that used genome-wide significant SNP(s) as a fixed effect(s) in the model. Individual SNP specific genetic variances estimated using the direct method (Table 4) should not be considered as an additive function because these QTL SNPs are in LD with an average value of ~ 0.30 (ranging from 0.05–0.65, Fig. 5). Moreover, the observation of high shrinkage in *P*-values for all the SNPs at linkage group 17 (Fig. 3a-b, Additional file 2: Table S2.5A-B) with the use of the most significant SNP (Locus ID: 248936_32) as a fixed effect gives an indication for the presence of single QTL in the region. Hence, the variances explained by the genome-wide significantly associated SNPs within this region could either be averaged (mean %***varG*** = 10.53, Table 4) or variance explained by the most significant SNP (Locus ID: 248936_32 and %***varG*** = 16.14, estimated in indirect method) should be the one accounted to avoid any possible inflations. The relatively large impact of single QTL on the total genetic variation (13.3% using Falconer and Mackay [59], and 16.14% applying the indirect method) does not necessarily mean that the tagged SNP is a causative mutation, but this SNP explains an important amount of QTL variation, either directly or through LD with the causative mutations.

The sequence (36 bp) of the highest significantly associated tag/SNP (248936_32) was aligned to the unpublished sea bream genome assembly (Saurata_v1), which resulted in significant (e-value = 4.00e-09) alignment with chromosome 19. The region of approximately ±50Kb surrounding the highest significant SNP “248936_32” was searched for underlying candidate genes that might affect the resistance against pathogen. Detected genes within the specified region are detailed in (Additional file 2: Table S2.6). The SNP aligned to a position 8,024,839 bp at chromosome 19 and the flanking region of ±50Kb contained 4 genes (Additional file 2: Table S2.6). *Sox-17 alpha-like* (*Sox-32*) is an *SRY-box* (*Sox*) transcription factor that is important during development of different tissues and organs. *Sox-17* has been recently reported to also control adult hematopoiesis [60, 61] . Considering that blood precursors differentiate into several lineages of immune-relevant cell types, it might be possible that genetic variants at *Sox-17* locus have a role in response to bacteria. The next gene encodes a mitochondrial protein that is part of the large (39S) ribosomal subunit, without clear association with immune response [62].

Acyl- thioesterase 1 (*ACT1*) is a key enzyme regulating an important post-translational protein modification, S-palmitoylation. Protein palmitoylation is crucial for functioning of key immune-related proteins (e.g. T cell receptor, Fcε receptor I, Fcγ receptor II, toll-like receptor 4). Bacteria might also high-jack the palmitoylation machinery of host cells to internalization, survival, and replication [63].

Regulator of G protein signaling 20 belongs to a family of regulator of G protein signaling (*RGS*) proteins, which are regulatory and structural components of G protein-coupled receptor (*GPCRs*) complexes [64]. It is known that *RGS* proteins expressed in immune effector cell such as mast cells and lymphocytes as well as in their end-organ targets (i.e., bronchial smooth muscle) represent an important regulatory component of the intracellular signaling pathways induced by *GPCRs* in allergic inflammation [64, 65]. Infectious agent (*Phdp*) of this disease has ability to cause septicemia and inflammation in different organs (e.g. liver, guts etc.). Variation in expression levels of *RGS* protein due to genetic variants at the locus may lead variation in resistance.

Based on available information, the most likely candidate genes appear to be *SOX-17* for its role in proliferation and differentiation of blood cells, *RGS* which has shown to play a role in allergic inflammation by activating G-protein-coupled receptors, and *ACT1* because palmitoylation has been shown to be important in bacterial infections.

Overall, the accuracy of prediction for resistance against *Phdp* using genomic information was observed to be 19 to 24% higher than the accuracy obtained using pedigree information which clearly shows that genomic information based predictions are much better than pedigree-based ones. A similar trend of higher accuracies using genomic- vs. pedigree-based information has been reported in Atlantic salmon [18, 28, 29] for parasite and pathogen resistance traits, as well as in other livestock species [66–68] on production traits. The advantage of genomic information over pedigree is because of realized genomic-based relatedness between animals deviate from pedigree-based relationship coefficients which can also utilize relationships among individuals which might not be related through the known pedigree. Genomic-based realized relationships also unravel within family variation which is not captured by pedigree information. In general, genomic selection in aquaculture populations allows predictions with higher accuracy especially for traits that cannot be measured on the selection candidates (e.g. disease resistance and fillet quality) and the potential within family can also be fully exploited with the use of genomic information.

Irrespective of source of information (pedigree or genomic) used, we observed 29 to 52% higher accuracy of prediction compared to the reported results by Palaiokostas et al. (2016) [9]. The PBLUP based accuracies (0.465–0.449; Table 5) obtained in our study were around 52% higher than the estimate of 0.30 obtained by Palaiokostas et al. (2016) [9]. Genomic information based average accuracy (0.557) obtained in this study was ~ 29% higher than the average genomic based accuracy of 0.43 reported by Palaiokostas et al. (2016) [9]. This discordance could be because of differences in population background (e.g. effective population size) and population structure (e.g. average relationship between training and validation) which might lead to better predictions and higher selection accuracies due to elevated LD in a specific population [67]. The obtained lower genomic accuracy of Palaiokostas et al. (2016) [9] compared to our results could partially be because of relatively smaller dataset on phenotypes (777 vs. 1187) as well as SNP markers (11,239 vs. 22,544).

The comparison of accuracy of prediction within genomic information based models (GBLUP, BayesB, BayesC, and Bayesian Lasso) showed that BayesB fractionally outperformed all other models for both *P*_*DS*_ and *P*_*D*2*D*_, and the ranking was followed by Bayesian Lasso BayesC and GBLUP with estimates as an average of both traits (*P*_*DS*_ and *P*_*D*2*D*_). Relative performance of the GS models depends on the genetic architecture of the trait, and the GWAS results of our analysis revealed that a few genes/loci with moderate to large effect and many loci with a small effect might be involved in total genetic variation of the trait. Thus, Bayesian prediction methods are expected to perform better or equally well as GBLUP method [29, 67] which was found concordant with the expectations.

## Conclusion

The current study demonstrates that SNP based genotyping of a sea bream population using 2b-RAD approach is effective at capturing the genetic variation for resistance against *Phdp*. A genome-wide significant QTL at LG17 was detected which explained 13.28 to 16.14% of genetic variation. The QTL region is encased by genes like *SAP, Il6ra* and 26S non-ATPase regulatory subunit which might be involved in variation for resistance against *Phdp*. Prediction accuracies obtained using genomic information were significantly higher (19 to 24%) than the accuracies obtained using pedigree information which highlights the importance and potential of genomic selection in commercial breeding programs.

## Additional files

Additional file 1: Information on inflation of *P*-value, LD among significant SNPs, genes at QTL region and Manhattan plots. This file contains information on average and sex-specific (male vs. female) linkage maps along with 36 bp sequences of 2b-RAD loci (XLSX 1344 kb).

Additional file 2: Information on inflation of *P*-value, LD among significant SNPs, genes at QTL region and Manhattan plots. This file comprises supporting information which contains Q-Q plot, zoom in view of GWAS based *P*-values distributed at chromosome 2, information on genes around the highest significant SNP of chromsome2, LD values with heatmap for the top 5 significant markers of GWAS analysis, and Manhattan plots showing SNP positions corrected using linkage map information, and the shrinkage of *p*-values using model with the addition of top significant SNP used as fixed effect. **Figure S2.1.** Distribution of dead and alive sibs out of total sib count per full-sib family. **Figure S2.2.** Manhattan plot of –log10 *P*-values for P_D2D trait distributed across different LGs. **Figure S2.3.** (A-B): Manhattan plot of –log10 *P*-values for P_D2D trait distributed along the length (cM) of SA17. **Figure S2.4.** Q Q-Plot of –log10 *P*-values – days to death phenotype (P_D2D). **Table S2.5.** Variances explained by genome-wide significantly associated SNPs of P_D2D trait. **Table S2.6.** Summary for the functions of important candidate genes at QTL region (DOCX 718 kb).

## Abbreviations

cM: CentiMorgans
FMD: Ferme Marine de Douhet
GWAS: Genome wide association studies
LG: Linkage groups
*P*_*D2D*_: Days to Death Phenotype
*P*_*DS*_: Dead/Survive phenotype
*Phdp*: *Photobacterium damselae* subsp*. piscicida*
QC: Quality controls
QTL: Quantitative trait locus
RAD-Seq: Restriction-site associated DNA sequencing
SNPs: Single nucleotide polymorphisms
