# Supplementary material for: Genetics of resistance to photobacteriosis in gilthead sea bream (Sparus aurata) using 2b-RAD sequencing
Source: BMC Genet. 2018 Jul 11;19:43. doi: 10.1186/s12863-018-0631-x (PMC6042378; doi:10.1186/s12863-018-0631-x)
Supplement: Supplementary file 2 — Information on inflation of P-value, LD among significant SNPs, genes at QTL region and Manhattan plots. This file comprises supporting information which contains Q-Q plot, zoom in view of GWAS based P-values distributed at chromosome 2, information on genes around the highest significant SNP of chromsome2, LD values with heatmap for the top 5 significant markers of GWAS analysis, and Manhattan plots showing SNP positions corrected using linkage map information, and the shrinkage of p-values using model with the addition of top significant SNP used as fixed effect. Figure S2.1. Distribution of dead and alive sibs out of total sib count per full-sib family. Figure S2.2. Manhattan plot of –log10 P-values for P_D2D trait distributed across different LGs. Figure S2.3. (A-B): Manhattan plot of –log10 P-values for P_D2D trait distributed along the length (cM) of SA17. Figure S2.4. Q Q-Plot of –log10 P-values – days to death phenotype (P_D2D). Table S2.5. Variances explained by genome-wide significantly associated SNPs of P_D2D trait. Table S2.6. Summary for the functions of important candidate genes at QTL region (DOCX 718 kb). [file 12863_2018_631_MOESM2_ESM.docx]

**Additional File 2:** Information on distribution of sibs in families, GWAS based results for $P_{D2D}$ trait along with description of underlying genes at QTL region.

**
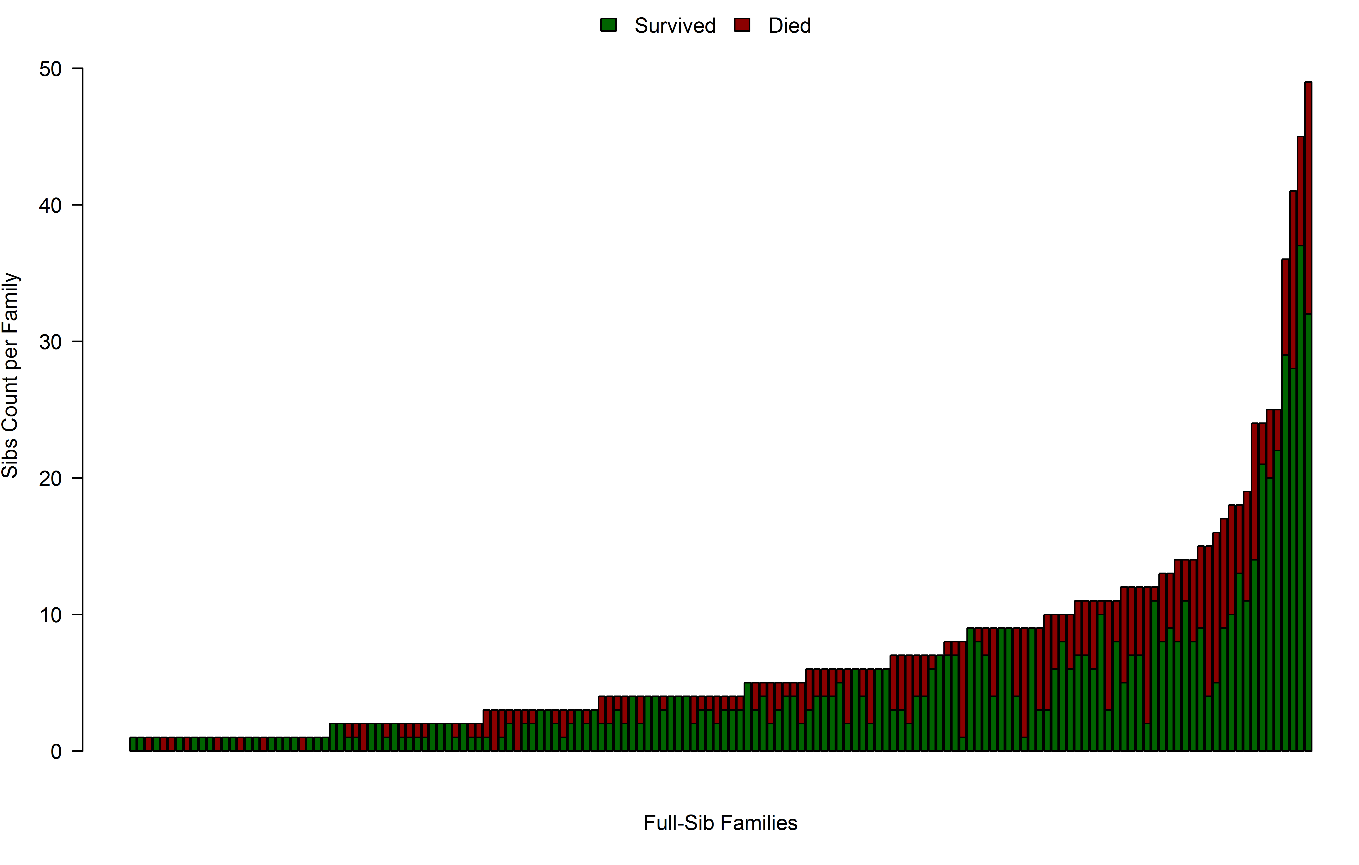
Figure S2.1: Distribution of dead and alive sibs out of total sib count per full-sib family**

**Figure S2.2: Manhattan plot of –log_10_ P-values for** $\boldsymbol{P}_{\boldsymbol{D}\boldsymbol{2}\boldsymbol{D}}$ **trait** **distributed** **across different LGs.**

**Figure S2.3(A-B): Manhattan plot of –log_10_ P-values for** $\boldsymbol{P}_{\boldsymbol{D}\boldsymbol{2}\boldsymbol{D}}$ **trait distributed along the length (cM) of SA17.**

Highlighted green dots represent genome-wide significant SNPs and horizontal solid line represents the Bonferroni significance threshold (–log_10_ P-values = 5.654). **A)** Plot with all the markers on SA17 and the top most significant SNP is highlighted green with asterisk (*) symbol; **B)** plot after correcting for the top most significant SNP and using it as a fixed effect in the model.

**Figure S2.4: Q**  **Q-Plot of –log_10_ P-values – days to death phenotype** $\boldsymbol{(}\boldsymbol{P}_{\boldsymbol{D}\boldsymbol{2}\boldsymbol{D}}\boldsymbol{)}$


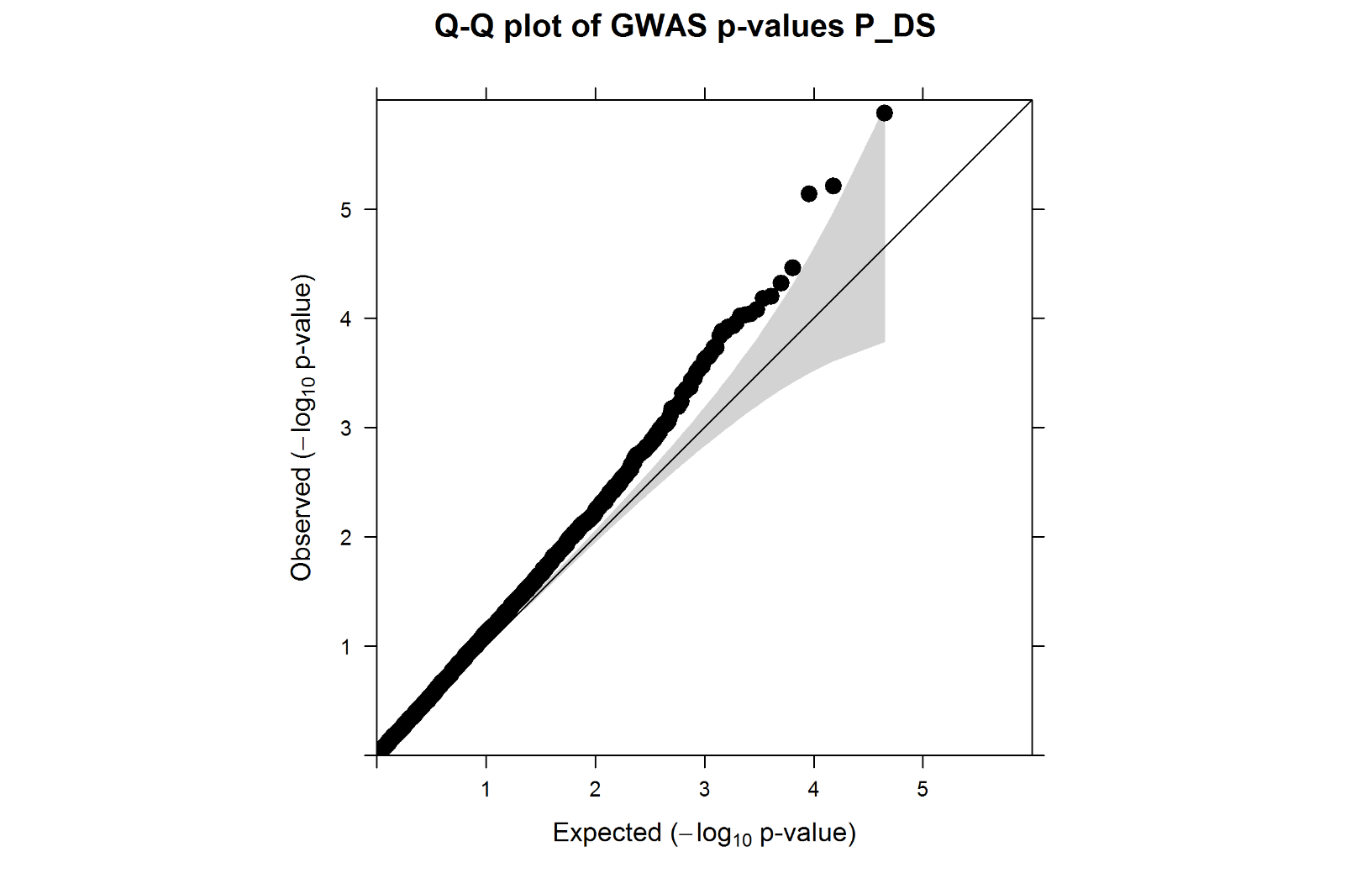


**λ= 1.132356**

**Table S2.5: Variances explained by genome-wide significantly associated SNPs of** $\boldsymbol{P}_{\boldsymbol{D}\boldsymbol{2}\boldsymbol{D}}$ **trait.**

| **SA** | **Locus ID** | **Pos** | **Allele1** | **Allele2** | **MAF** | $\boldsymbol{\alpha}$ | **SE** | **P-value** | $\boldsymbol{\%}\boldsymbol{varG}$ | $\boldsymbol{\%}\boldsymbol{varP}$ |
| --- | --- | --- | --- | --- | --- | --- | --- | --- | --- | --- |
| 17 | 248936_32 | 44.729 | G | T | 0.278 | 2.051 | 0.339 | 1.39E-09 | 13.411 | 4.257 |
| 17 | 24643_19 | 49.430 | A | G | 0.467 | 1.771 | 0.299 | 3.00E-09 | 12.385 | 3.931 |
| 17 | 283885_36 | 44.372 | G | C | 0.343 | 1.767 | 0.302 | 4.98E-09 | 11.169 | 3.545 |
| 17 | 211563_28 | 53.066 | A | G | 0.353 | 1.848 | 0.334 | 3.06E-08 | 12.384 | 3.931 |
| 17 | 81173_9 | 40.766 | A | G | 0.362 | 1.640 | 0.306 | 8.14E-08 | 9.859 | 3.130 |
| 17 | 265801_18 | 37.385 | T | C | 0.321 | 1.694 | 0.324 | 1.77E-07 | 9.929 | 3.152 |
| 17 | 95842_16 | 51.835 | G | C | 0.425 | -1.476 | 0.296 | 6.12E-07 | 8.448 | 2.682 |
| 17 | 53157_6 | 44.333 | T | C | 0.179 | 1.959 | 0.407 | 1.46E-06 | 8.930 | 2.835 |
| 17 | 80727_35 | 44.216 | A | C | 0.211 | 1.768 | 0.370 | 1.80E-06 | 8.263 | 2.623 |
| 17 | 3001_36 | 48.242 | A | G | 0.238 | 1.712 | 0.360 | 1.98E-06 | 8.449 | 2.682 |
| 17 | 225775_29 | 40.582 | T | C | 0.367 | -1.515 | 0.319 | 2.04E-06 | 8.461 | 2.686 |

SA = linkage group number for gilthead seabream (*Sparus aurata, SA*); Pos(cM) = genetic map position of SNP; A1 & A2 = minor & major alleles, respectively; MAF = minor allele frequency; α = Allele substitution effect; SE = standard error; P = significance value; $varG$= proportion of genotypic variance explained; $varP$ = proportion of phenotypic variance explained.

**Table S2.6: Summary for the functions of important candidate genes at QTL region.**

| **Genes (around ± 50Kb)** | **Description** | **Ref** |
| --- | --- | --- |
| *transcription factor Sox-17-alpha-like* | Sox-17 and Sox-17 alpha-like (Sox-32) are SRY-box (Sox) transcription factors that are important during development of different tissues and organs. Sox-17 has been recently reported to control also adult hematopoiesis which is s a formation of blood cellular components including immune cell components of blood. | [1, 2] |
| *39S ribosomal mitochondrial protein* | mitochondrial protein that is part of the large (39S) ribosomal subunit. | [3] |
| *Acyl- thioesterase 1* | Acyl- thioesterase 1 (ACT1) is a key enzyme regulating an important post-translational protein modification, S-palmitoylation. Protein palmitoylation is crucial for functioning of key immune-related proteins (e.g. T cell receptor, Fcε receptor I, Fcγ receptor II, toll-like receptor 4) | [4] |
| *regulator of G- signaling 20* | RGS proteins expressed in immune effector cell such as mast cells and lymphocytes as well as in their end-organ targets (i.e., bronchial smooth muscle) represent an important regulatory component of the intracellular signaling pathways induced by GPCRs in allergic inflammation. | [5, 6] |

**REFERENCES**

1. Maha A, Ikuo N, Mitsujiro O, Atsushi I, Kaho H, Kiyoka S, Tetsuya T: **Sox17 as a candidate regulator of myeloid restricted differentiation potential**. *Development, Growth & Differentiation* 2014, **56**(6):469-479.

2. Cuvertino S, Lacaud G, Kouskoff V: **SOX7-enforced expression promotes the expansion of adult blood progenitors and blocks B-cell development**. *Open Biology* 2016, **6**(7).

3. Koc EC, Burkhart W, Blackburn K, Moyer MB, Schlatzer DM, Moseley A, Spremulli LL: **The Large Subunit of the Mammalian Mitochondrial Ribosome: ANALYSIS OF THE COMPLEMENT OF RIBOSOMAL PROTEINS PRESENT**. *Journal of Biological Chemistry* 2001, **276**(47):43958-43969.

4. Sobocińska J, Roszczenko-Jasińska P, Ciesielska A, Kwiatkowska K: **Protein Palmitoylation and Its Role in Bacterial and Viral Infections**. *Frontiers in Immunology* 2017, **8**:2003.

5. Druey KM: **Regulation of G-protein-coupled signaling pathways in allergic inflammation**. *Immunologic research* 2009, **43**(1-3):62-76.

6. Druey KM: **Chapter Nine - Emerging Roles of Regulators of G Protein Signaling (RGS) Proteins in the Immune System**. In: *Advances in Immunology.* Edited by Shukla AK, vol. 136: Academic Press; 2017: 315-351.
